# Associations between polygenic risk, negative symptoms, and functional connectome topology during a working memory task in early-onset schizophrenia

**DOI:** 10.1038/s41537-022-00260-w

**Published:** 2022-06-02

**Authors:** Mengjie Deng, Zhening Liu, Wen Zhang, Zhipeng Wu, Hengyi Cao, Jie Yang, Lena Palaniyappan

**Affiliations:** 1 Department of Psychiatry, The Second Xiangya Hospital of Central South University, Changsha, Hunan China; 2 National Clinical Research Center for Mental Disorders, Changsha, Hunan China; 3 Center for Psychiatric Neuroscience, Feinstein Institutes for Medical Research, Manhasset, NY USA; 4 Division of Psychiatry Research, Zucker Hillside Hospital, Glen Oaks, New York, NY USA; 5 Douglas Mental Health University Institute, Department of Psychiatry, McGill University, Montreal, Quebec Canada; 6 Department of Medical Biophysics, Schulich School of Medicine and Dentistry, Western University, London, Ontario Canada; 7 Department of Psychiatry, Schulich School of Medicine and Dentistry, Western University, London, Ontario Canada; 8 Robarts Research Institute, Schulich School of Medicine and Dentistry, Western University, London, Ontario Canada

**Keywords:** Neuroscience, Developmental biology

## Abstract

Working memory (WM) deficit in schizophrenia is thought to arise from a widespread neural inefficiency. However, we do not know if this deficit results from the illness-related genetic risk and influence the symptom burden in various domains, especially in patients who have an early onset illness. We used graph theory to examine the topology of the functional connectome in 99 subjects (27 early-onset schizophrenia (EOS), 24 asymptomatic siblings, and 48 healthy subjects) during an n-back task, and calculated their polygenic risk score (PRS) for susceptibility to schizophrenia. Linear regression analysis was used to test associations of the PRS, clinical symptoms, altered connectomic properties, and WM accuracy in EOS. Indices of small-worldness and segregation were elevated in EOS during the WM task compared with the other two groups; these connectomic aberrations correlated with increased PRS and negative symptoms. In patients with higher polygenic risk, WM performance was lower only when both the connectomic aberrations and the burden of negative symptoms were higher. Negative symptoms had a stronger moderating role in this relationship. Our findings suggest that the aberrant connectomic topology is a feature of WM task performance in schizophrenia; this relates to higher polygenic risk score as well as higher burden of negative symptoms. The deleterious effects of polygenic risk on cognition are played out via its effects on the functional connectome, as well as negative symptoms.

## Introduction

Schizophrenia is a multigene disorder with high heritability^1,2^, with more than 145 independent genomic risk loci being associated with this illness in genome-wide association studies (GWAS)^3,4^. The cumulative effect of these genes is thought to contribute to the phenotype of core symptoms and cognitive deficits seen in patients. A polygenic risk score (PRS) based on the GWAS results was developed to measure the additive effects of multiple risk genes^5–7^. The genetic risk for schizophrenia varies among patients based on the age of illness onset; higher genetic risk burden is thought to hasten the age of onset, so early-onset patients display higher genetic burden than patients with adulthood onset schizophrenia^8,9^.

The unaffected siblings of patients with schizophrenia share 50% genetic background with patients^10^. They share certain endophenotypes that are often subtle, nonclinical features such as cognitive impairment^11^, neurological soft signs^12^, and brain structural and functional abnormalities^13^. Compared with patients, the unaffected siblings were free of antipsychotics and chronic disease course influence. Therefore, investigating these endophenotypes in unaffected siblings may help to acknowledge the clinical or biological findings that are more closely related to risk genes than the disorder itself.

As a key feature of schizophrenia, working memory (WM) deficit is also an important endophenotype of schizophrenia^14^. The WM deficit not only has been observed in patients with schizophrenia throughout the prodromal stage as well as the psychotic phase in the disease, which persists even after symptomatic remission resulting in a lifelong cognitive burden, but also has been observed in their unaffected siblings^15,16^. Substantial research on the heritability of neurocognition has estimated that the heritability of working memory is around 33–64%^17–19^. Furthermore, previous studies^20,21^ probing the neural mechanism of WM impairment in schizophrenia and their siblings have revealed the continuity or a similar pattern of neural abnormities between these two groups. However, as with some siblings who have subtle symptoms, it is difficult to distinguish whether these neural abnormities that merely related to genetic risks or also associated with these subtle clinical symptoms.

The neural inefficiency phenomenon during WM performance has been widely endorsed in schizophrenia^22^. There is a left shift of the inverted U-shaped model of WM-related cortical engagement in schizophrenia^23^. Previous studies have indicated that patients tend to adopt a compensating neural strategy to facilitate the improvement of WM performance at higher loads but this energy-hungry process is not sustainable, driving up the burden of negative symptoms^23,24^. Negative symptoms have been demonstrated to affect the WM performance and mediate the relationship between WM deficits and functional outcome^25–27^. In our prior work, we investigated the neural efficiency of the whole-brain functional connectome during the WM task in schizophrenia by employing graph theory tools^28^. Patients showed a more homogeneous network organization compared with HCs, but with elevated modular segregation of topologically proximal brain regions rather than the global integration that is critical for the WM task^29^. This inefficiently reconfigured pattern of the whole-brain functional connectome in schizophrenia replicated across different datasets with heterogeneous clinical data and robust across different parcellation schemes. However, we do not know if this aberrant connectome configuration is also seen in siblings that are unaffected and relates to PRS of schizophrenia.

In the current study, we aim to explore whether the inefficient connectome origination pattern emerges in siblings and whether this pattern is genetically or clinically related to the disease. To this end, we employed the graph theory methods to investigate the connectome organization of patients with early-onset schizophrenia (EOS) and their siblings during WM tasks. We also calculated their PRS scores to identify the intermediate neurophysiological phenotypes that may be affected by these cumulative genetic effects, which may help to better understand the functional role of these genetic variations. The linear regression model was adopted to probe the association between the PRS, clinical symptoms, connectome topology, and WM performance. To the best of our knowledge, this is the first study assessing the functional connectome profile in the EOS and their siblings during WM task, and further probing the relationship between this profile with the genetic risks, clinical symptoms, and WM performances.

## Results

### Participant characteristics

As shown in Table 1, one-way analysis of variance revealed a significant omnibus difference in education *(F*_2,98_ = 5.0, *p* = 0.0085), and accuracy of WM under 2-back load (*F*_2,89_ = 8.42, *p* = 0.0005) across all groups, but no significant omnibus difference in age (*F*_2,98_ = 1.85, *p* = 0.16) and gender (*χ*^*2*^ = 4.03, *p* = 0.133). Post hoc tests revealed the education years of HCs were longer than that of EOS (*p* = 0.0026), and the accuracy of WM under 2-back load of HCs was higher than that of EOS (*p* = 0.0001) and SB (*p* = 0.049).

**Table 1:** Demographic, neuropsychological, and clinical data.

Items	EOS (*n* = 27)	SB (*n* = 24)	HCs (*n* = 48)	*F*/*χ*^*2*^	*P* value	Post Hoc Significance
Age (Years)	18.37 (0.63)	19.92 (0.67)	19.73 (0.47)	1.89	0.16	N/A
Gender (M/F)	14/13	8/16	28/20	4.03	0.133	N/A
Education (Years)	10.63 (0.5)	11.5 (0.5)	12.5 (0.37)	5.0^a^	0.0085^a^	EOS < HCs; *p* = 0.0026;
Illness duration (Month)	25.6 (17.7)	N/A	N/A	N/A	N/A	N/A
Total dosage (mg)	399.84 (342.2)	N/A	N/A	N/A	N/A	N/A
PANSS_total	65.89 (25.05)	N/A	N/A	N/A	N/A	N/A
PANSS_N	17.89 (9.2)	N/A	N/A	N/A	N/A	N/A
PANSS_P	13.67 (7.4)	N/A	N/A	N/A	N/A	N/A
PANSS_G	30.8 (13.4)	N/A	N/A	N/A	N/A	N/A
PANSS_S	3.52 (1.3)	N/A	N/A	N/A	N/A	N/A
SAPS	22.2 (21.1)	N/A	N/A	N/A	N/A	N/A
SANS	43.7 (35.2)	N/A	N/A	N/A	N/A	N/A
ACC_2back	0.7 (0.14)	0.77 (0.18)	0.84 (0.12)	8.55^a^	0.0004^a^	EOS < HCs; *p* = 0.0001; SB < HCs; *p* = 0.049;
ACC_0back	0.88 (0.16)	0.87 (0.2)	0.94 (0.09)	2.53	0.09	N/A
RTC_2back (ms)	709.6 (150.7)	715.1 (154.5)	638.1 (124.5)	3.02	0.054	N/A
RTC_0back (ms)	532.6 (115.2)	548.1 (86.6)	496.2 (78.4)	2.6	0.08	N/A

*n* number, *PANSS* Positive and Negative Syndrome Scale, *PANSS_N* the sum score of all negative items in the Positive and Negative Syndrome Scale, *PANSS_P* the sum score of all positive items in the Positive and Negative Syndrome Scale, *PANSS_G* the sum score of all general items in the Positive and Negative Syndrome Scale, *PANSS_S* the sum score of all supplemental items in the Positive and Negative Syndrome Scale, *SAPS* the Scale for the Assessment of Positive Symptoms, *SANS* the Scale for the Assessment of Negative Symptoms, *N/A* not available. *ACC_2*back accuracy under the 2-back load, *ACC_0*back accuracy under the 0-back load, *RTC_2*back response time under the 2-back load, *RTC_0*back response time under the 0-back load, *EOS* early-onset schizophrenia, *SB* siblings, *HCs* healthy controls.

^a^Significantly different among three groups.

### Genetic data

In our current study, due to the limited number of subjects, there was no significant omnibus difference in the synthesized PRS across all three groups (EOS mean (SD) = 25.63 (70.7), SB mean (SD) = 21.52 (84.37), HCs mean (SD) = −30.28 (96.4)).

### Network properties

**Fig. 1:**
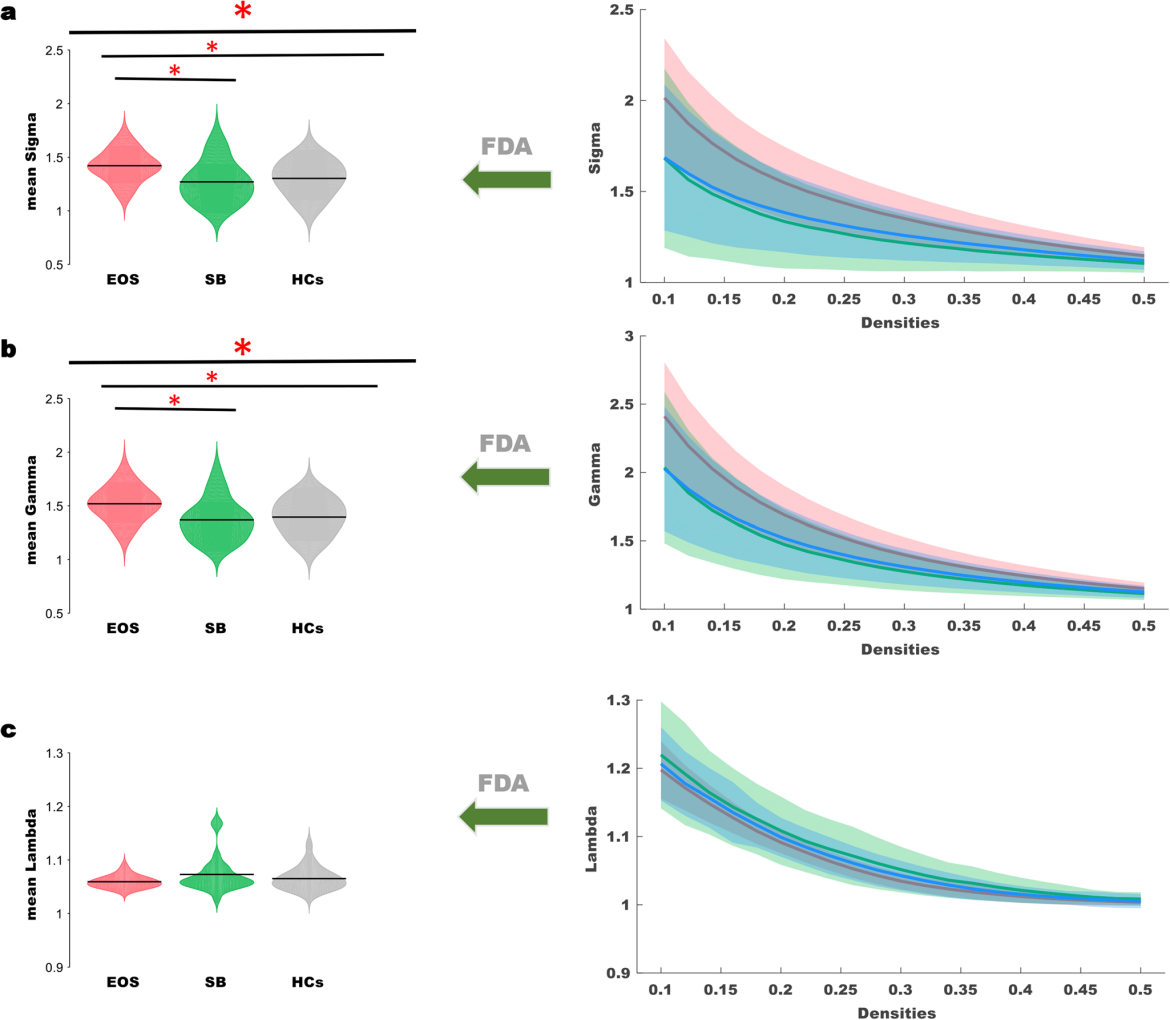
Global properties of the whole-brain functional connectome calculated on Power atlas in three diagnostic groups. The range of densities is 0.1:0.02:0.5, and symbol “*” represents *p* < 0.05. **a** Comparison of mean sigma across densities among three groups; **b** comparison of mean gamma across densities between three groups; **c** comparison of mean lambda across densities among three groups.

One-way analysis of variance revealed a significant omnibus difference in sigma across all diagnostic groups (EOS mean (SD) = 1.421(0.031), SB mean (SD) = 1.27(0.033), HCs mean (SD) = 1.3 (0.023), *F*_2,98_ = 6.66, *p* = 0.002, see Fig. 1a and Table 2). Post hoc tests revealed significantly increased sigma in EOS compared to SB (*p* = 0.0012), as well as HCs (*p* = 0.0029).

**Table 2:** Global network measures showing significant omnibus alteration.

Items	EOS (*n* = 27)	SB (*n* = 24)	HCs (*n* = 48)	*F*/*χ*2	*P* value	Post Hoc Significance
Sigma	1.421 (0.031)	1.27 (0.033)	1.3 (0.023)	6.61^a^	0.002^a^	EOS > SB; *p* = 0.0012; EOS > HCs; *p* = 0.0029;
Gamma	1.52 (0.032)	1.37 (0.034)	1.39 (0.024)	6.66^a^	0.002^a^	EOS > SB; *p* = 0.0015; EOS > HCs; *p* = 0.0024;
Lambda	1.06 (0.004)	1.073 (0.005)	1.065 (0.0033)	2.25	0.11	N/A

*n* number, *EOS* early-onset schizophrenia, *SB* siblings, *HCs* healthy controls, *HCs* healthy controls.

Sigma or small-worldness is the ratio of gamma (normalized clustering coefficient) over lambda (normalized characterized path length).

^a^Significantly different among three groups.

One-way analysis of variance indicated a significant omnibus difference in gamma across all diagnostic groups (EOS mean (*SD*) = 1.52(0.032), SB mean (*SD*) = 1.37(0.034), HCs mean (*SD*) = 1.39 (0.024), *F*_2,98_ = 6.6, *p* = 0.002, see Fig. 1b). Post hoc tests revealed significantly increased gamma in EOS compared with SB (*p* = 0.0015), as well as HCs (*p* = 0.0024).

The omnibus alteration of lambda was not observed across all diagnostic groups (EOS mean (SD) = 1.06(0.004), SB mean (SD) = 1.073(0.005), HCs mean (SD) = 1.065 (0.0033), *F*_2,98_ = 2.25, *p* = 0.11, see Fig. 1c).

At the regional level, we did not observe significant omnibus difference in the regional clustering coefficient across all diagnostic groups after multiple comparison corrected.

### Exploratory analysis

#### Correlation analysis

**Fig. 2:**
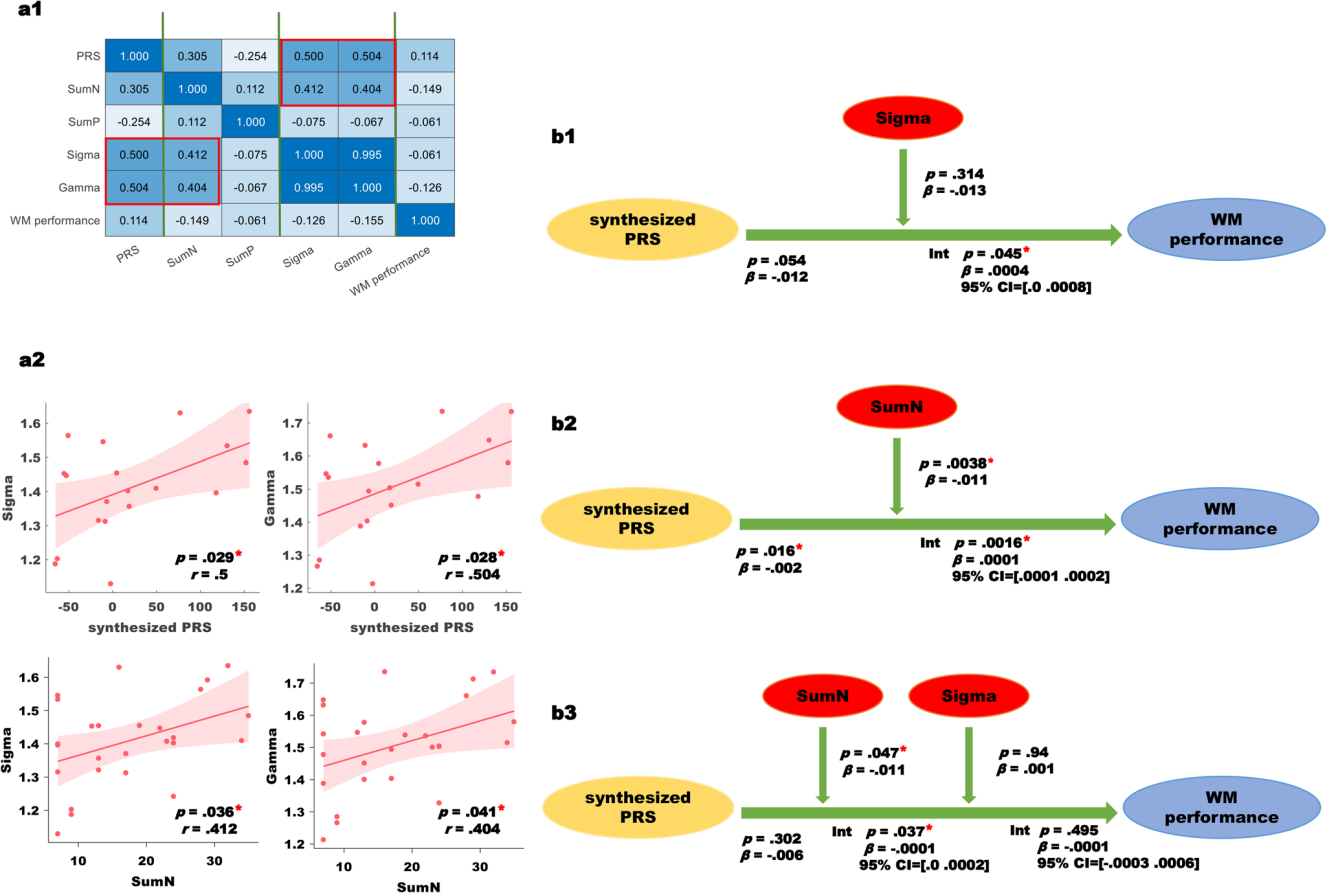
Exploratory analysis. The symbol “*” represents *p* < 0.05. **a** Correlation analysis among synthesized PRS, clinical symptoms (including SumN [negative symptom scores] and SumP [positive symptom scores]), altered network measures (including sigma and gamma), and WM performance; **b** Linear regression analysis among the synthesized PRS, negative symptoms, sigma, and WM performance.

We observed the significant correlation between the synthesized PRS with the altered global network measures—sigma (*p* = 0.029; *r* = 0.5) and gamma (*p* = 0.028; *r* = 0.504), as well as the significant correlation between the negative symptoms with the altered global network measures—sigma (*p* = 0.036; *r* = 0.412) and gamma (*p* = 0.041; *r* = 0.404). There were no association among other parameters (see Fig. 2a1 and a2).

#### Moderation analysis

We were interested in exploring whether reduced WM performance in EOS results from the illness-related genetic risk (PRS), and if the symptom burden and/or connectomic aberrations influence this relationship. As our linear regression analysis indicated a significant relationship between the connectomic measures and negative symptoms as well as synthesized PRS, we explored a chain of moderation models to test the association between the synthesized PRS, a static measure as the independent variable and negative symptoms, and altered global network measures—both of which are variable over time—as mediators to predict WM performance (dependent variable). As shown in Fig. 2b1 and b2, in EOS, sigma moderated the relationship between synthesized PRS and WM performance (*p* = 0.045, *β* = 0.0004); and negative symptoms moderated the relationship between synthesized PRS and WM performance (*p* = 0.0016, *β* = 0.0001). Then, we constructed a moderation model (model 2 in PROCESS), and set both the negative symptoms and sigma as moderator variables. As shown in Fig. 2b3, in EOS, we observed the also negative symptoms (*p* = 0.037, *β* = −0.0001), but not the sigma (*p* = 0.495, *β* = −0.0001), moderated the relationship between synthesized PRS and WM performance. When we treated the gamma as a moderator variable, we observed results similar to those described above (see Supplemental material S2).

## Discussion

To our knowledge, this is the first study combining the sibling and genetic risk data to demonstrate functional connectome topology changes associated with WM deficits in EOS. We report three main findings. Firstly, there is a significant increase in small-worldness in EOS compared with their siblings and HCs; this increase is driven by the local clustering but not the global integration. Secondly, these connectomic aberrations are associated with an increase in polygenic risk and negative symptoms. Thirdly, the polygenic risk influenced the WM performance through connectomic aberrations and negative symptoms, with negative symptoms being a more influential moderator of the polygenic risk -WM performance relationship than the connectome topology.

We observed that patients with EOS showed an increased small-worldness compared with siblings and HCs, and this increase was attributed to the elevated segregation but with no concomitant increase in integration. The current findings are consistent with our previous observations^28^ which supported the increased small-worldness accompanied by the elevated segregation in three datasets across early-onset, adult-onset, and chronic schizophrenia during the WM task. As connectome-level functional integration is seen as critical for working memory^29^, the increased segregation that we observed during the WM task may reflect a neural integrative inefficiency in schizophrenia; thus, the observed neural activity cannot effectively and sustainably promote cognitive performance, especially at higher loads.

There were no group differences between siblings and HCs in network measures with the omnibus alteration. Contrary to our findings, previous studies have demonstrated the continuity (i.e., a similar pattern) of neural abnormalities among patients with schizophrenia and siblings during the WM task. For example, Landin-Romero et al.^20^ have documented that both the patients and the siblings showed a significant failure of deactivation in the medial frontal cortex compared with the HCs; Loeb et al.^21^ have reported that siblings had altered functional activation and connectivity intermediate between patients with childhood-onset schizophrenia and controls in the frontoparietal and cortico-striatal regions. Nonetheless, previous studies also reported inconsistent findings especially when the sibling group was more carefully separated. Choi et al.^30^ have observed that, during the WM task, healthy first-degree relatives of patients (genetic high risk or GHR) showed increased activation but the ultra-high-risk groups with prodromal symptoms (UHR) and patients showed decreased activation in the frontoparietal network compared with HCs. In that study, the ordinal pattern of the frontoparietal network activation was GHR > HCs>UHR ≈ patients. Therefore, the observed continuity (i.e., a similar pattern) of neural deficits among patients with schizophrenia and siblings may associate with the subtle clinical symptoms in the recruited siblings. In the context of Choi et al. study^30^, we speculate that the altered functional connectome topology associated with WM deficits in schizophrenia may relate more to clinical expression rather than genetic burden.

In the EOS, the increased small-worldness and segregation correlated with increased PRS and negative symptoms, and these network measures and negative symptoms can separately moderate the association between PRS and WM performance. We observed that increased small-worldness and segregation have a positive effect on WM performance in patients with higher genetic risks. This finding resonates with our prior study^28^ that demonstrated the more severely affected patients (e.g., higher genetic risk or more severe clinical symptoms), can make modest improvements in their WM performance only when higher physiological efforts are spent, but the accuracy level thus achieved would still be considerably lower than what the healthier individuals achieve with less effort. Here we find higher genetic risk in those with more serious negative symptoms improved better WM performance modestly. Considering the negative symptoms positively correlated with the network properties, we speculate this performance promotion effect of polygenic factors may indeed reflect the higher physiological efforts (i.e., processing cost) in EOS. In fact, in the multivariate moderation analysis, when variance due to connectome aberrations was accounted for, PRS in patients with higher negative symptoms related to reducing WM performance, i.e., not “performance-enhancing” anymore. These constellations may indicate that the genetic risk has an effect on the WM performance through the negative symptoms and functional connectome organization, and the negative symptoms have a critical important moderating role, exerted in part, through the functional connectome topology.

## Limitations

There are several limitations in the present study. First, despite their suitability for sibling-paired samples, there are many challenges in recruiting EOS subjects; this study was limited to a single site and we lacked another independent dataset to testify the replication of our study. Second, we did not find significant difference in PRS among EOS, SB, and HCs, which may be a type-2 error. Future studies with larger sample size and replication dataset will be needed to further validate our findings. We urge the readers to treat these as hypothesis-forming observations for future confirmation. Finally, as all patients in our study were treated with antipsychotics and their illness duration was not restricted when they were recruited, we urge caution when attempting to generalize these findings to untreated cohorts.

## Conclusions

In the present study, we demonstrate that the architecture of the functional connectome is altered in early-onset schizophrenia during a WM task, and this abnormality tracks the burden of negative symptoms. The polygenic risk for schizophrenia influences cognitive performance indirectly by increasing the negative symptom burden, as well as through its effects on the functional connectome. In the presence of higher polygenic risk, efforts to address negative symptoms may have more impact on subsequent cognitive rehabilitation.

## Methods

### Participants

The procedures of the present study were approved by the medical ethics committee of the Second Xiangya Hospital, Central South University, Changsha, China. Prior to obtaining consent, two licensed psychiatrists with at least 2 years of working experience in psychiatry department ascertained the capacity of all potential participants to provide informed consent. All participants were right-handed native Chinese and they signed written informed consent prior to study enrollment. All study procedures were in strict accordance with the Declaration of Helsinki.

A total of 39 patients with early-onset schizophrenia (EOS) and 33 of their unaffected siblings (SB) were recruited from the Second Xiangya Hospital of Central South University. Under the assessment of clinical psychiatrists, all patients met the schizophrenia criteria of Structured Clinical Interview of DSM-IV (SCID) and the clinical symptoms of patients were assessed by using the Positive and Negative Symptom Scale (PANSS)^31^, the Scale for the Assessment of Positive Symptoms (SAPS)^32^ and the Scale for the Assessment of Negative Symptoms (SANS)^33^. The exclusion criteria for patients with EOS were: (1) age <14 or >45 years old; (2) the age of first onset >18 years old; (3) history of alcohol or substance dependence; (4) neurological disorders, former recorded brain injury or physical diseases; (5) history of receiving electroconvulsive therapy; (6) any contraindications for magnetic resonance imaging (MRI) scanning; (7) benzodiazepine treatment, if any, stopped for at least 24 h before scanning. The exclusion criteria for SB were the same as those for patients except that they did not meet the DSM-IV criteria for any mental disorders.

Fifty-six age- and sex-matched healthy controls (HCs) were recruited from the local community and were assessed using the SCID non-patient edition. The exclusion criteria for HCs were the same as those for patients except that they did not meet the DSM-IV criteria for any mental disorders and their first-degree relatives had no history of any known mental disorders.

### MRI data acquisition and pre-processing

All neuroimaging data were obtained on a Siemens Allegra 3-T scanner with a gradient-recalled echo-planar imaging pulse sequence. And the parameters were showed as followed: repetition time (TR) = 2000 ms, echo time (TE) = 30 ms, flip angle = 90°, FOV = 240 × 240 mm^2^, acquisition matrix = 64 × 64, slices = 32, slice thickness = 5 mm, gap =0 mm, and total volumes = 253.

Imaging data were preprocessed and analyzed by the DPABI toolbox^34^. The first five images were discarded for scanner stabilization, and a total of 248 volumes were obtained for preprocessing. The remaining volumes were slice-time corrected, realigned to correct for head motion, spatially normalized into the brain template of Montreal Neurologic Institute (MNI), and smoothed (FWHM = 8 mm). Linear detrending was performed and followed by nuisance covariates regression including 12 head motion parameters (including derivatives), white matter, and CSF signals. As recent research has shown illness-related variance in the global signals^35^, the global signal was not removed. Displaced volumes (framewise displacement >0.5 mm) were interpolated by nearest-neighbor interpolation^36,37^. Samples were excluded if they met the following criteria: (1) head motions >2.5 mm translation or >2.5° rotation; (2) fMRI data visually examined by experienced data analysts that failed to normalize to MNI space. After quality control, a total of 28 EOS, 25 SB, and 48 HCs were included in the final analysis. No significant difference was found in framewise displacement (total number of interpolated volumes) across all three groups (EOS mean(SD) = 7.88(11.09), SB mean(SD) = 8.33(11.48), HCs mean(SD) = 9.28(13.9), *F*_2,98_ = 0.11, *p* = 0.89).

### Genetic data processing

We collected whole blood samples of all subjects and extracted genomic DNA by the EZgene Blood gDNA Miniprep Kit. Next, we performed whole-genome genotyping by using the standard Illumina genotyping protocol on Illumina Asian Screening Array (ASA) Bead Chip. All genetic data underwent standard quality control using PLINK 1.9^38^. We removed the sample with higher missing genotype rate in each pair that more similar genotypes were identified by calculating the pairwise identity-by-descent than we would expect in a random sample, removing 1 individual from the dataset of EOS and 1 individual from the dataset of SB. And a total of 27 EOS, 24 SB, and 48 HCs were left in the present study. Next, a principal component analysis (PCA) was conducted to control the population stratification using EIGENSTART^39,40^ on a linkage disequilibrium (LD) pruned set of autosomal SNPs obtained by carrying out LD pruning with PLINK and removing five long-range LD regions with the Hap Map phase 3 reference dataset^41^. Finally, we excluded the SNPs with missing genotype rates <0.95, a minor allele frequency (MAF) < 0.01, or a significant departure from Hardy-Weinberg Equilibrium (HWE, *P* < 1.0 × 10^−6^), leaving about 396,343 SNPs.

Genotypes after quality control were performed imputations for non-genotyped genetic variants using the SHAPEIT^42^ and IMPUTE^43^ with the Phase 3 multi-ethnic 1000 Genomes Projects panel, as the reference based on the human genome assembly hg19 (https://mathgen.stats.ox.ac.uk/impute/1000GP_Phase3.html). We removed SNPs with imputation quality control <0.8, missing genotyping rate <0.95, MAF < 0.01, or significant departure from HWE (*P* < 1.0 × 10^–6^). About 7,672,168 SNPs survived the pruning procedures and were used to calculate the PRS score.

The PRS analysis was performed using the PRSice toolbox^44^ based on the GWAS results from the Psychiatric Genomics Consortium (https://www.med.unc.edu/pgc/results-and-downloads). All matched SNPs between the base and target datasets were clumped based on the LD threshold of *R*^2^ < 0.2 within a 500 kb window. The scores were computed as the total of genome-wide risk alleles for each participant, weighted by the corresponding odds ratios to schizophrenia. We calculated PRSs with a set of thresholds [5 × 10^−8^; 5 × 10^−5^; 5 × 10^−5^; 0.1; 0.1001:1 × 10^−4^; 0.5 1] (6002 PRS in total). We used the PCA method to do dimension reduction, and retrieve the first component (explained 89.3% of total variance) to represent the synthesized PRS.

### Network construction

The WM paradigm adopted in the present study comprised two load conditions (0-back and 2-back, the details were given in Supplementary material S1). To construct the process of functional connection matrix, only the fMRI volumes obtained in the four blocks of 2-back performance were concatenated^45^, as the 0-back load is not considered a task of WM. We extracted the mean time series from each of the 264 nodes with 6 mm spheres defined by the Power atlas^46^. We calculated the Pearson correlation coefficients of the time series for each pair of ROIs to generate a 264 × 264 symmetric matrix was for each participant and applied the Fisher z transformation to convert the resultant matrix into normally distributed scores. We also controlled the variance caused by the linear effects of demographic data including age, gender, and education years to derive the corrected symmetric matrix.

With using scripts from the Brain Connectivity Toolbox (http://www.brain-connectivity-toolbox.net/), we calculated global network measures including sigma (small-worldness), lambda (normalized characterized path length), gamma (normalized clustering coefficient), and regional network measure—clustering coefficient (strongly associated with gamma) on the 264 × 264 weighted adjacency matrices at a series of network densities (0.1:0.02:0.5). The sigma is a ratio of gamma to lambda (ie., sigma = gamma/lambda). The normalized topological properties—gamma and lambda, must be benchmarked against corresponding mean values of null random graphs (i.e., gamma = *C*/*C*_*null*_ and lambda = *L*/*L*_*null*_, where *C* indicates the clustering coefficient, and *L* indicates the path length). Wherein, we generated 20 null random networks, with the same number of nodes, degree, and degree distribution as the network of interest.

### Statistical analysis

We applied the one-way analysis of variance (ANOVA) and *χ*^2^ tests to detect group differences in demographic, neuropsychological characteristics, and behavioral data. As for network metrics, we employed the functional data analysis (FDA)^47^ to synthesize values across densities. In the FDA, each network metric curve is treated as a function (*y* = f(*x*)), and the sum of differences in *y*-values is calculated across densities. Then, the synthesis network metrics were subsequently allowed to the one-way ANOVA analysis, and followed by the post hoc comparison procedure when significant main effects were present. Furthermore, we adopted the Benjamini-Hochberg method (FDR corrected) with *p* < 0.05 for multiple comparison to generate statistical maps of regional network metrics.

### Exploratory analysis

#### Correlation analysis

We conducted the correlation analysis between any two of the four types of parameters, including the synthesized PRS, altered global network properties, clinical symptoms (positive and negative symptoms), and WM performance.

#### Moderation analysis

We adopted linear regression analysis to test the association of the synthesized PRS, WM performance, clinical symptoms, and altered global network properties in the EOS. The linear regression analysis was conducted on the PROCESS 3.0 macro that was embedded in SPSS_22_, with a 5000 bias-corrected bootstrap sample for significance testing.

## Supplementary information

Supplemental material

## Data Availability

The data that support the findings of this study are available from the corresponding author upon reasonable request.
