# Supplementary material for: Associations between polygenic risk, negative symptoms, and functional connectome topology during a working memory task in early-onset schizophrenia
Source: Schizophrenia (Heidelb). 2022 Jun 2;8(1):54. doi: 10.1038/s41537-022-00260-w (PMC9261080; doi:10.1038/s41537-022-00260-w)
Supplement: Supplementary file 1 — Supplemental material [file 41537_2022_260_MOESM1_ESM.docx]

**S1. Detailed descriptions of the N-back task.**

The N-back task was conducted on the Nordic Neurolab’s functional magnetic resonance imaging (fMRI) hardware system and lasted for 8 minutes and 16 seconds. Initially, there was 4 seconds as a short-fixed time before the working memory (WM) task started to calibrate MRI scanner. Then, a blocked design of tasks was presented with four 40 seconds blocks of the “0-back” condition, alternating with four 40 seconds blocks of the “2-back” condition. Each block was preceded by a task instruction for 2 seconds. After each task block, a fixed period of 20 seconds was inserted to provide a recovery time for hemodynamic response between task blocks. The data from these fixation periods were also used as the pre-stimulus baseline (condition “rest”). All stimuli were sequences of white capital letters on a black background, presented in pseudo-random order at the center of the screen (500 ms duration, 1500 ms inter-stimulus interval).

Participants were instructed to respond to each stimulus by using a 2-button response box with one for the target letters and the other for the non-target letters. Under the 2-back condition, when the current letter matched the letter that appeared two trials prior, it was identified as ‘target’, and vice versa. The examples of ‘target’ stimulus under 0-back and 2-back conditions were represented in the Figure S1. The task performance (response time and accuracy) of each participant, was recorded electronically by a computer connected to fMRI.


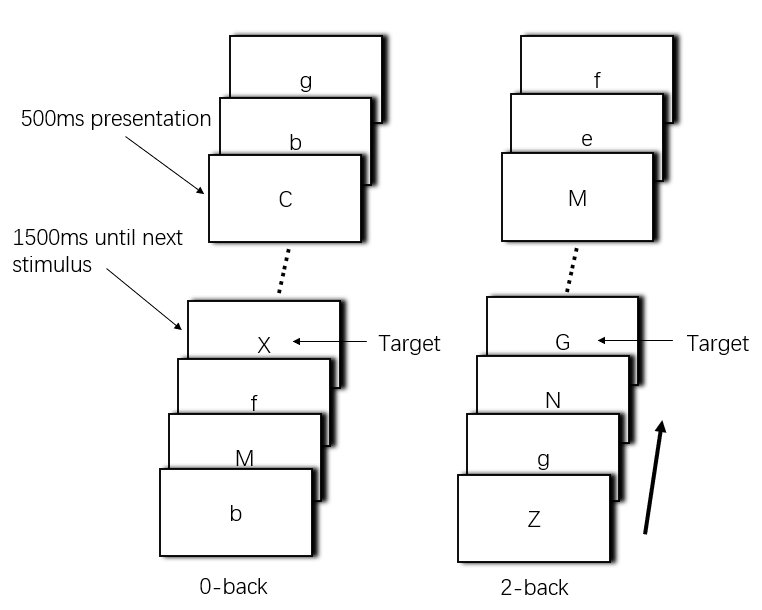


**Figure S1** The ‘target’ stimulus under 0-back and 2-back conditions.

**S2. Detailed descriptions of the moderation analysis.**

We observed the significant correlation between the synthesized PRS with the altered global network measures — sigma (*p*=0.029; *r*=0.5) and gamma (*p*=0.028; *r*=0.504), as well as the significant correlation between the negative symptoms with the altered global network measures — sigma (*p*=0.036; *r*=0.412) and gamma (*p*=0.041; *r*=0.404). There were no association between other parameters (see Figure S2[a1] and Figure S2[a2]).

As shown in Figure S2[b1] and Figure S2[b2], in EOS, gamma moderated the relationship between synthesized PRS and WM performance (*p*=0.046, *β*=0.0004); and negative symptoms moderated the relationship between synthesized PRS and WM performance (*p*=0.0073, *β*=0.0001). Then, we constructed a moderation model (the model 2 in PROCESS), and set both the negative symptoms and gamma as moderating variables. As shown in Figure S2[b3], in EOS, we observed the also negative symptoms (*p*=0.04, *β*=-0.0001), but not the gamma (*p*=0.46, *β*=-0.0001), moderated the relationship between synthesized PRS and WM performance.


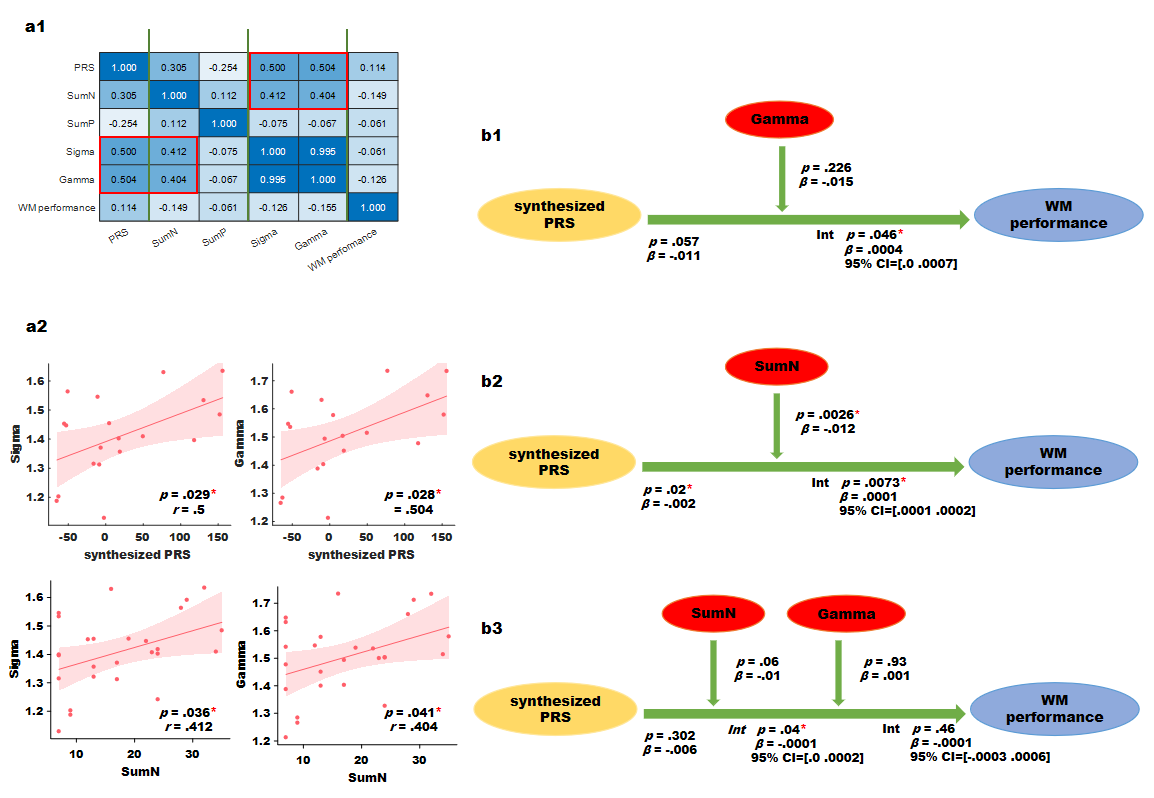


**Figure S2 Exploratory analysis.** (a) Correlation analysis among synthesized PRS, clinical symptoms (including SumN [negative symptom scores] and SumP [positive symptom scores]), altered network measures (including sigma and gamma), and WM performance; (b) Linear regression analysis among the synthesized PRS, negative symptoms, gamma, and WM performance.
